# Individualized prediction of survival benefit from postoperative radiotherapy for patients with malignant pleural mesothelioma

**DOI:** 10.1002/cam4.5955

**Published:** 2023-04-19

**Authors:** Yang Wo, Yizhou Peng, Zhigang Wu, Pengcheng Liu, Yan Shang, Xuxia Shen, Yihua Sun

**Affiliations:** ^1^ Department of Thoracic Surgery and State Key Laboratory of Genetic Engineering Fudan University Shanghai Cancer Center Shanghai China; ^2^ Institute of Thoracic Oncology Fudan University Shanghai China; ^3^ Department of Oncology, Shanghai Medical College Fudan University Shanghai China; ^4^ Department of Pathology Fudan University Shanghai Cancer Center Shanghai China

**Keywords:** nomogram, prognostic factor, radiotherapy, SEER

## Abstract

**Objectives:**

The role of postoperative radiotherapy (PORT) in malignant pleural mesothelioma (MPM) remains controversial and the eighth edition TNM staging scheme for MPM has not been fully verified. We aimed to develop an individualized prediction model for identifying optimal candidates for PORT among MPM patients who received surgery plus chemotherapy and externally validate the performance of the new TNM staging scheme.

**Materials and Methods:**

Detailed characteristics of MPM patients during 2004–2015 were retrieved from SEER registries. Propensity score matching (PSM) was conducted to reduce disparities of baseline characteristics (age, sex, histologic type, stage, and type of surgery) between the PORT group and no‐PORT group. A novel nomogram was constructed based on independent prognosticators identified by multivariate Cox regression model. The discriminatory performance and degree of calibration were evaluated. We stratified patients into different risk groups according to nomogram total scores and estimated the survival benefit of PORT in different subgroups in order to identify the optimal candidates.

**Results:**

We identified 596 MPM patients, among which 190 patients (31.9%) received PORT. PORT conferred significant survival benefit in the unmatched population, while there was no significant survival difference favoring PORT in the matched population. The C‐index of the new TNM staging scheme was closed to 0.5, which represented a poor discriminatory ability. A novel nomogram was constructed based on clinicopathological factors, including age, sex, histology, and N stage. We stratified patients into three risk groups. Subgroup analyses indicated that PORT was beneficial for high‐risk group (*p* = 0.003) rather than low‐risk group (*p* = 0.965) and intermediate‐risk group (*p* = 0.661).

**Conclusion:**

We established a novel predictive model, which could make individualized prediction of survival benefit of PORT for MPM and could compensate for weakness in TNM staging system.

## 1 INTRODUCTION

Malignant pleural mesothelioma (MPM) is a rare malignancy predominantly involving the pleural surface, with approximately 80%–90% diagnosed cases suffering from asbestos exposure.^1^, ^2^ The past few decades witnessed a dramatic increase in the incidence of MPM, and it is estimated that around 40,000 cases will die of asbestos‐related mesothelioma per year.^3^ Due to the lack of credible screening tool and effective treatment guideline, MPM is characterized by extremely poor survival, with a median survival of 12–18 months.^4^

In 2016, the International Association for the Study of Lung Cancer (IASLC) proposed revisions for the eighth TNM staging system for MPM based on detailed study of multi‐institutional databases and clinical trials. Major modifications introduced for the new staging system included the following: (1) collapse of T1a/1b into a unified T1 category, (2) grouping previous N1 and N2 categories into a single N1 category, (3) redefining N3 as N2 category, and (4) revisions of stage groups.^5^, ^6^, ^7^, ^8^ However, the prognostic performance of new TNM staging scheme has been questioned by the IASLC Mesothelioma Staging Project and has not been fully verified in large series; thus, external validation and further refinement of the current staging criteria is necessary.

Although surgical interventions remain the mainstay of treatment for early‐stage MPM, the postoperative care of MPM necessitates multidisciplinary partnerships because surgery alone is insufficient to improve the quality of care.^9^ Postoperative radiotherapy (PORT) has been identified as a component of multidisciplinary management for MPM, potential benefits of which include the ability to improve local control and facilitate parenchymal‐sparing surgical resection.^10^ However, owing to the rarity of the disease and the lack of evidence from large prospective trials,^11^ the role of PORT in MPM patients who received surgery plus chemotherapy remains controversial and proper administration of PORT is particularly demanding, which renews the interest in evaluating the benefits and indications of PORT. We hypothesized that multiple clinicopathological factors would combine to influence the treatment benefit of PORT in MPM patients and disaggregation of the overall outcomes in accordance with risk levels would allow for more individualized estimation of PORT benefit. In this study, we aimed to develop an individualized survival prediction model based on clinicopathological factors and identify the optimal candidates for PORT among MPM patients using the Surveillance, Epidemiology, and End Results (SEER) database. In addition, we also externally validated the eighth TNM staging system for MPM using SEER datasets and compared the prognostic performance of traditional TNM staging scheme and individualized prediction model.

## 2 MATERIALS AND METHODS

### 2.1 Study cohorts

Detailed characteristics of MPM patients during 2004–2015 were retrieved from the SEER 18 registries according to the following eligibility criteria: (1) pathologically confirmed MPM with ICD‐O‐3 codes 9050–9053, 9055 and primary site code C38.4; (2) treatment with cancer‐directed surgery and chemotherapy; (3) without distant metastasis; and (4) active follow‐up. Patients with history of preoperative radiotherapy, unspecified TNM stage, and missing baseline characteristics were excluded. Patients were reclassified according to the eighth TNM staging system for MPM. Since the SEER database was publicly accessible, approval from Institutional Review Board was not required.

### 2.2 Statistics

Categorical variables were presented as frequency (percentage) and were assessed with the Pearson *χ* ^2^ test and Fisher's exact test. To reduce disparities of baseline characteristics (age, sex, histologic type, stage, and type of surgery) between the PORT group and no‐PORT group, we therefore conducted propensity score matching (PSM) using *MatchIt* package in R with a 1:1 nearest neighbor matching. Overall survival (OS), defined as the interval from diagnosis to death from any factors, was the endpoint variable and was estimated with Kaplan–Meier methods and log‐rank tests. Nomogram was constructed using *rms* and *survival* packages in R software based on independent prognostic indicators determined by univariate and multivariate Cox regression models. The discriminatory power and calibration of prediction models were measured by Harrell's C‐index and calibration plots, respectively, with a better model indicated by greater C‐index and favorable consistency between the predicted and actual survival. Internal validation of the developed model was conducted through bootstrap (*B* = 1000) resamples. To better clarify the benefit of PORT in patients with distinct clinicopathological factors, we therefore stratified patients into several risk groups according to total risk scores calculated by developed prediction model. The optimal cut points to determine risk group classifications were defined by X‐tile program.^12^ We further estimate the benefit of PORT in different risk groups and identified the optimal candidates for PORT. Statistical analyses were conducted with SPSS 22.0 (IBM, USA) as well as R 3.5.3 (R Project, Austria). Statistical significance was declared with a two‐tailed *p* value <0.05.

## 3 RESULTS

### 3.1 Baseline characteristics

**FIGURE 1:**
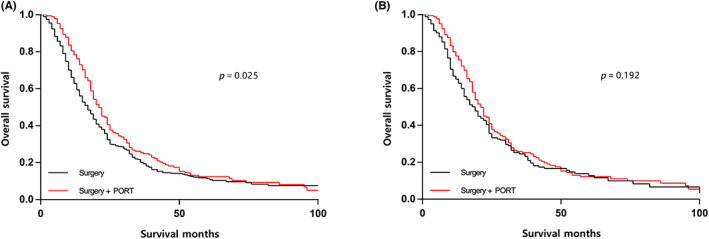
Survival curves of overall survival according to treatment modality in the unmatched (A) and matched (B) populations. PORT, postoperative radiotherapy.

A total of 596 qualified cases with MPM diagnosed between 2004 and 2015 were enrolled (Table 1), among which 190 patients (31.9%) underwent trimodality treatment and 406 patients (68.1%) received surgery plus chemotherapy. The mean age of enrolled patients was 65.3 years, and male (79.5%) outnumbered female (20.5%) patients. Significant discrepancies in clinicopathological factors were observed between the patients who received and who did not receive PORT before PSM. Specifically, patients who received PORT were more likely to be younger, to be diagnosed with epithelioid histology, to have advanced N stage, and to complete radical surgery. Consequently, we conducted PSM and 184 pairs stratified by the choice of PORT were well matched (Table 1). PORT conferred significant survival benefit in the unmatched population (median OS: 17 vs. 21 months; *p* = 0.025; Figure 1A), while there was no significant survival difference favoring PORT in the matched population (median OS: 19 vs. 21 months; *p* = 0.192; Figure 1B).

**TABLE 1:** Patients’ characteristics.

Variable	Before matching			After matching		
	no‐PORT (*n* = 406)	PORT (*n* = 190)	*p* value	no‐PORT (*n* = 184)	PORT (*n* = 184)	*p* value
Age			<0.001			0.902
<60	80 (19.7)	65 (34.2)		58 (31.5)	61 (33.2)	
60–70	185 (45.6)	92 (48.4)		90 (48.9)	90 (48.9)	
>70	141 (34.7)	33 (17.4)		36 (19.6)	33 (17.9)	
Histologic type			0.016			0.688
Epithelioid	220 (54.2)	122 (64.2)		114 (62.0)	116 (63.0)	
Biphasic	67 (16.5)	33 (17.4)		29 (15.8)	33 (17.9)	
Sarcomatoid	119 (29.3)	35 (18.4)		41 (22.2)	35 (19.1)	
Sex			0.498			0.613
Female	80 (19.7)	42 (22.1)		42 (22.8)	38 (20.7)	
Male	326 (80.3)	148 (77.9)		142 (77.2)	146 (79.3)	
T stage			0.309			0.949
T1	81 (19.9)	28 (14.7)		26 (14.1)	28 (15.2)	
T2	107 (26.4)	46 (24.2)		40 (21.7)	43 (23.4)	
T3	132 (32.5)	73 (38.4)		75 (40.8)	70 (38.0)	
T4	86 (21.2)	43 (22.7)		43 (23.4)	43 (23.4)	
N stage			0.041			0.249
N0	262 (64.5)	106 (55.8)		95 (51.6)	106 (57.6)	
N1–2	144 (35.5)	84 (44.2)		89 (48.4)	78 (42.4)	
Surgery			<0.001			0.601
Radical	121 (29.8)	88 (46.3)		87 (47.3)	82 (44.6)	
Palliative	285 (70.2)	102 (53.7)		97 (52.7)	102 (55.4)	

Abbreviation: PORT, postoperative radiotherapy.

### 3.2 External validation of the eighth TNM staging system

**FIGURE 2:**
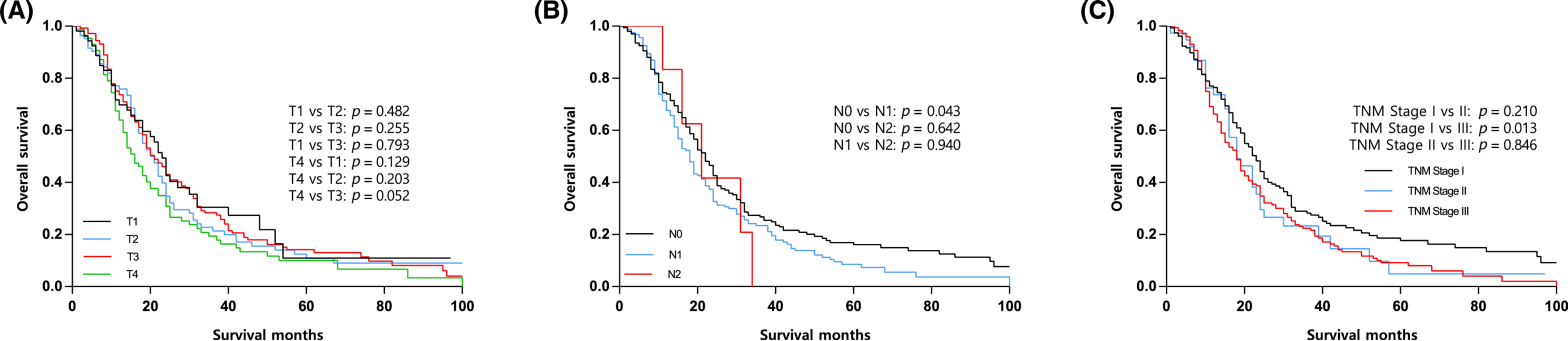
Survival curves of overall survival according to (A) T descriptors, (B) N descriptors, and (C) TNM stage. PORT, postoperative radiotherapy.

Subgroup analyses of T descriptors of eighth TNM staging system indicated that the survival differences between adjacent T descriptors did not achieve statistical significance (Table 2; Figure 2A). N descriptors exhibited an uneven distribution and there was a significant difference in OS between stage N0 and N1 MPM patients (median OS: 22 vs. 18 months; *p* = 0.043; Figure 2B). The majority of cases were classified as N0 (54.6%) and N1 (43.8%), with only 1.6% classified as N2. Thus, we failed to define the prognosis of N2 disease owing to limited cases within that category, and we merged N1 and N2 into a single category in the subsequent Cox regression analyses (Table 2). Moreover, the eighth TNM staging failed to demonstrate significant survival difference in stage I versus II (*p* = 0.210; Figure 2C) as well as stage II versus III (*p* = 0.846; Figure 2C).

**TABLE 2:** Cox regression analysis of independent prognostic factors.

Variable	Univariate		Multivariate	
	HR (95% CI)	*p* value	HR (95% CI)	*p* value
Age		<0.001		0.001
<60	1		1	
60–70	1.639 (1.259–2.134)	<0.001	1.551 (1.187–2.026)	0.001
>70	1.851 (1.329–2.578)	<0.001	1.767 (1.264–2.471)	0.001
Sex		<0.001		0.001
Male	1		1	
Female	0.603 (0.454–0.801)	<0.001	0.622 (0.467–0.829)	0.001
Histologic type		0.001		<0.001
Sarcomatoid	1		1	
Epithelioid	0.592 (0.437–0.800)	0.001	0.578 (0.426–0.784)	<0.001
Biphasic	0.850 (0.593–1.218)	0.376	0.918 (0.640–1.318)	0.643
T stage		0.221		
T1	1			
T2	1.129 (0.763–1.669)	0.544		
T3	1.053 (0.734–1.510)	0.780		
T4	1.391 (0.944–2.050)	0.095		
N stage		0.045		0.007
N0	1		1	
N1–2	1.259 (1.005–1.577)	0.045	1.375 (1.092–1.732)	0.007
Surgery		0.833		
Palliative	1			
Radical	0.976 (0.780–1.222)	0.833		
PORT		0.201		
No	1			
Yes	0.864 (0.691–1.081)	0.201		

Abbreviations: CI, confidence interval; HR, hazard ratio; PORT, postoperative radiotherapy.

### 3.3 Development of the prediction model

**FIGURE 3:**
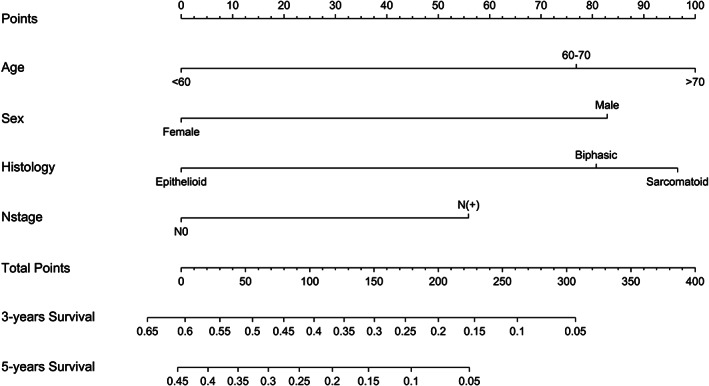
Constructed nomogram for predicting overall survival and benefit of postoperative radiotherapy in stage I–III malignant pleural mesothelioma.

In univariate Cox regression analysis (Table 2), clinicopathologic and baseline parameters that demonstrated a significant association with OS included age (*p* < 0.001), gender (*p* < 0.001), histologic subtype (*p* = 0.001), and N stage (*p* = 0.045). All of the above‐determined parameters were further integrated into the multivariate Cox regression models, which indicated that age (*p* = 0.001), gender (*p* = 0.001), histologic subtype (*p* < 0.001), and N stage (*p* = 0.007) were independent prognosticators for OS (Table 2). A nomogram for patients with stage I–III MPM was therefore developed based on independent prognostic factors including age, gender, histologic subtype, and N stage (Figure 3). Every descriptor of integrated parameters was designated a risk score on the point scale. Subsequently, the total risk scores of MPM patients, ranging from 0 to 400, could be calculated. Then, users were capable of visually estimating the 3‐ and 5‐year OS by drawing a straight line from the top total points scale down to the bottom 3‐ and 5‐year OS probability scales. Based on the measure of C‐index, the discriminatory power of the novel nomogram [C‐index = 0.656; 95% confidence interval (CI), 0.623–0.688] outperformed that of conventional TNM staging system (C‐index = 0.525; 95% CI, 0.494–0.556; *p* < 0.001). Calibration plots indicated an optimal concordance between the model‐predicted and actual observed 3‐ and 5‐year OS (Figure S1).

### 3.4 Identification of optimal candidates for PORT

**FIGURE 4:**
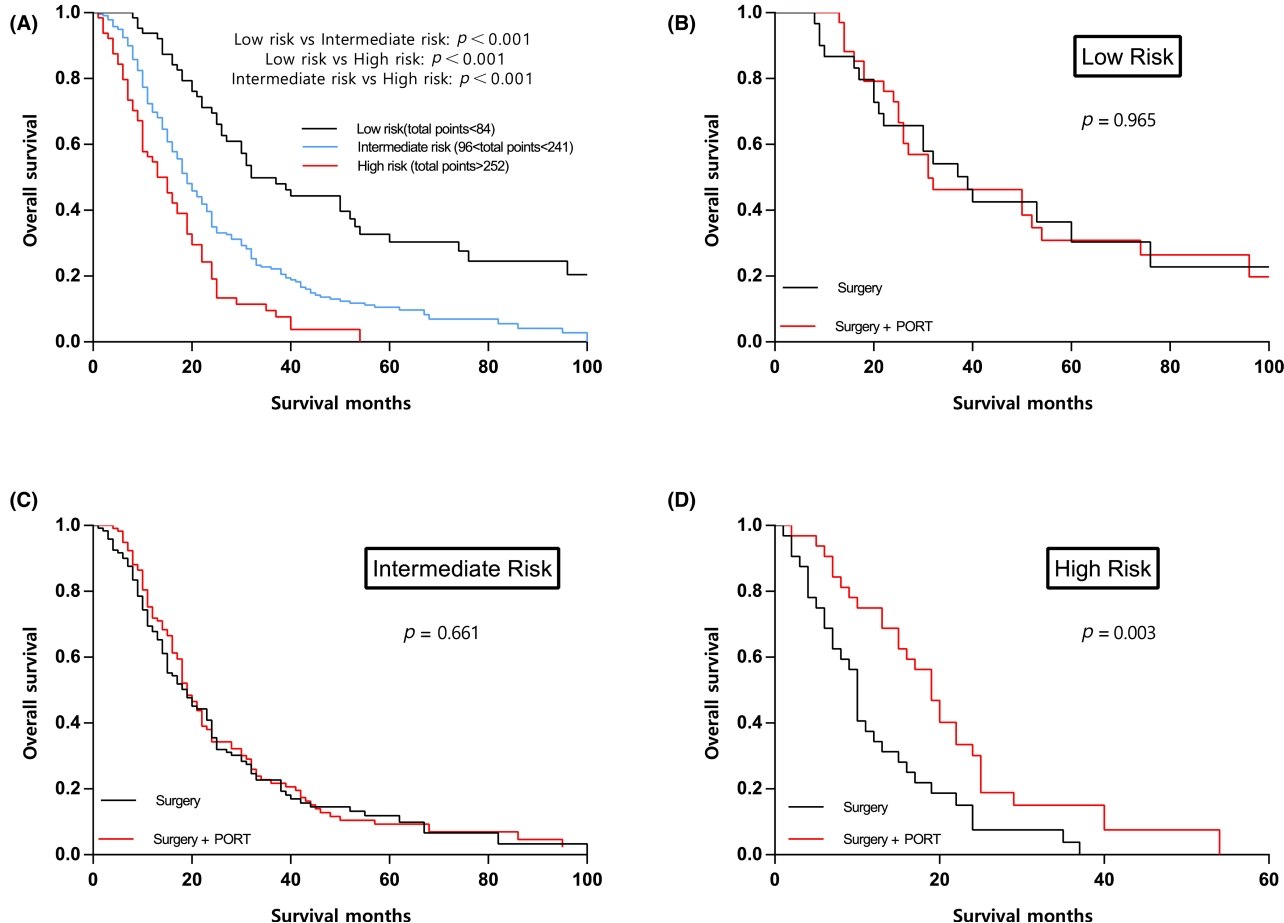
Survival curves of overall survival according to (A) risk subgroups, (B) treatment modality in low‐risk subgroup, (C) treatment modality in intermediate‐risk subgroup, and (D) treatment modality in high‐risk subgroup. PORT, postoperative radiotherapy.

Based on cut‐point study utilizing X‐tile, patients’ risk subgroups were stratified as follows: high risk (score > 252), intermediate risk (96 < score < 241), and low risk (score < 84). As shown in Figure 4A, the survival rate dramatically decreased with increasing risk level, and comparisons between adjacent risk subgroups demonstrated significant differences. In order to identify optimal candidates for PORT, we evaluated survival difference between patients who received and who did not receive PORT in each risk subgroup. We found that PORT significantly prolonged OS in high‐risk (*p* = 0.003; Figure 4D) subgroup, but not in low risk (*p* = 0.965; Figure 4B) and intermediate‐risk (*p* = 0.661; Figure 4C) subgroups. For example, for a 70‐year‐old (point = 76.9) female patient (point = 0) diagnosed as biphasic MPM (point = 80.7) with positive lymph nodes (point = 55.9), the model predicts that addition of PORT could not prolong survival (total points = 213.5; intermediate risk).

## 4 DISCUSSION

In this study, we established a nomogram based on clinicopathological parameters using SEER registry to predict OS and identify optimal candidates for PORT. We found that PORT significantly prolonged OS for high‐risk (score > 252) MPM patients. Moreover, we conducted an external validation of the prognostic performance of the eighth TNM staging system in resected MPM. Our study indicated that the latest TNM staging system which was modified based on robust survival data and sophisticated statistical analysis failed to precisely distinguish survival. The prognostic superiority of the novel prediction model over traditional TNM staging scheme has also been verified.

Data regarding benefits of conducting PORT for MPM patients remain heterogeneous. In several institutional studies, PORT has been identified to improve local control and prolong survival after resection of MPM.^13^, ^14^, ^15^ However, patients who receive surgery and chemotherapy are usually too moribund to undergo PORT or have quickly worsening disease, which might cause potential bias. One recent randomized trial focusing on the role of PORT in MPM is the Swiss SAKK 17/04 trials.^16^ In the SAKK study, patients received extrapleural pneumonectomy (EPP) following neoadjuvant chemotherapy, and those with macroscopic resection (R0/R1) were then randomized to PORT versus no radiation. Overall, the two treatment groups had similar locoregional control, and therefore, the investigators did not recommend the routine administration of PORT. Nevertheless, considering sizable patient dropout and lack of radiation guideline details, it is likely that the SAKK study might be underpowered to identify a difference.^17^ In this study, PORT conferred significant survival benefit in the unmatched population, while there was no significant survival difference favoring PORT in the matched population. In addition, when stratified by risk subgroup, PORT could only prolong survival in the high‐risk subgroup. Although results from randomized trials contribute robust evidence that whether a specific intervention works for proportions of enrolled patients, investigators need to be aware of how multiple clinicopathological factors might combine to influence the treatment benefit. We believe that MPM patients have multiple combinations of clinicopathological factors and could be stratified into different risk subgroups. If treatment effects vary across risk subgroups, then the overall summary results could not be generalized to all‐comers. Our customized prediction model is more relevant to individualized treatment planning compared with conclusions derived from coarse groupings of heterogeneous cases. Because SEER database did not record detailed radiotherapy data, we failed to comprehensively analyze the adverse events of PORT in MPM patients. The 2020 ERS/ESTS/EACTS/ESTRO and 2018 ASCO guidelines both stated that PORT could be offered to MPM patients preferably in experienced centers with exquisite technique and overall conditions of MPM patients should be fully assessed.^18^, ^19^ Therefore, apart from evaluating potential survival benefit, patient overall condition requires detailed assessment in treatment decision‐making process, and active follow‐up is warranted.

Surgical interventions play a crucial role in obtaining sufficient diagnostic samples, accurate staging, and multimodality treatment of MPM.^9^ We found that type of surgical procedures (palliative vs. radical surgery) was not independent prognosticator, and patients who underwent radical surgery were more likely to receive PORT, which could be attributed to the better overall condition in those who received radical resection. Detailed information regarding EPP, pleurectomy decortication (PD), and residual tumor classification was not available in SEER database. Therefore, we could only classify cancer‐directed surgery into radical or palliative surgery and failed to compare EPP with PD. Lapidot and colleagues showed that macroscopic complete resection (MCR) was associated with improved survival,^20^ which is consistent with several previous publications,^21^, ^22^ but in contrast with others.^23^, ^24^ Although the significance of achieving MCR remained controversial, there is no denying that the primary goal of cancer‐directed surgery should involve obtaining MCR. Moreover, the NCCN guidelines also emphasized the significance of surgical MCR. We believe that the logic for MCR is obvious. In addition, the surgical approach to a less radical lung‐sparing technique (PD) may improve OS compared with invasive EPP.^25^, ^26^, ^27^ Our study also demonstrated that histologic subtype had great impact on survival, and epithelioid subtype formed a sizable majority and was associated with better survival compared with biphasic and sarcomatoid subtypes, which is consistent with previous studies.^28^, ^29^, ^30^, ^31^ Meyerhoff et al. confirmed that surgical intervention was correlated with improved prognosis in epithelioid subtype but not in biphasic and sarcomatoid subtypes.^31^ Other guidelines also concluded that patients with sarcomatoid MPM were not optimal candidate for surgery.^18^, ^19^ Due to the long latent stage, the majority of diagnosed cases are aged 70 and older.^1^ The incidence of elderly MPM patients has increased sharply,^32^, ^33^ and proper management of those patients is particularly important. Yang and colleagues identified that surgery was associated with worse survival in patients aged 80 and older, which could be attributed to higher incidence of comorbidity and perioperative morbidity.^34^ In this study, patients with sarcomatoid subtypes accounted for 20.7% of the total, which reflected that a great number of patients who were not potential surgery candidates underwent surgery, and thus, MPM surgical treatment strategies require further refinement.

The TNM classification was developed to facilitate comprehensive evaluation of the anatomical disease extent. The IASLC MPM staging project collected 851 cases with pathologic lymph nodes (LNs) status from 29 international institutions.^6^ Based on the evidence that previous N1 and N2 descriptors were not correlated with different survival, IASLC recommended regrouping ipsilateral intrapleural and extrapleural LNs as N1 and reclassifying previous N3 as N2. In the SEER cohort, there was a significant difference in OS between stage N0 and N1 MPM patients. With regard to N stage distribution, only six cases (1.6%) were classified as N2, and we failed to define the prognosis of N2 disease owing to limited cases within that category. A similar uneven distribution of N stage was also observed in the IASLC staging dataset, which merely identified seven pathologic N2 cases and excluded them from prognostic studies. Clinical N descriptors in IASLC dataset, however, were also incapable of discriminating survival among adjacent descriptors, as survival curve of clinical N2 was superimposed with that of N0 and N1. Consistent with two recent publications focusing on outcomes of biphasic MPM,^21^, ^23^ we found that N stage was not predictive of survival in nonepithelioid MPM (Figure S2D–F). MPM histologic subtypes differ in that nonepithelioid MPM spread less frequently into LN than epithelioid MPM (34.8% vs. 51.7%; *p* = 0.002). Both the IASLC staging project and our external validation study highlighted the inaccuracy of MPM N category, which was derived from lung cancer LNs map. In fact, significantly distinctive LNs drainage pathways exist in MPM and lung cancer. The mesothelioma might involve diaphragm, mediastinal fat, pericardium, chest wall, thoracic vascular, and mediastinal fat, which makes the lymphatic drainage pattern even more complicated.^35^, ^36^, ^37^ However, there was no guideline regarding the technique and extent of LNs examination. In IASLC staging dataset, only about 10% of cases underwent pericardial, internal mammary, peridiaphragmatic, intercostal, and retrocrural LNs examination, which reflected extremely limited data availability on LNs metastatic pattern and variability in LNs sampling technique among thoracic surgeons.^6^ We recommend extensive LNs sampling during surgery in order to expand the understanding of LNs drainage pathway and disease involvement pattern of different histologic subtypes. For those who are not eligible for surgery, utilization of invasive sampling techniques such as mediastinoscopy and endobronchial ultrasound is advocated to enhance clinical LNs staging accuracy. Development of noninvasive predictive models based on image data would also be helpful. The key change of eighth T descriptors was to group T1a and T1b into one category (T1).^5^ However, orderly decrease in survival was not maintained in T categories, and there was no significant difference in survival between each T descriptor in SEER cohort. Similarly, in the IASLC Staging Project, no survival differences were noted from the pathologic staging between any of the T categories other than T3 and T4.^5^ This result was also observed in several previous analysis.^21^, ^23^ However, a recent study identified T descriptor as one of the most significant prognostic factors, which might justify the eighth T categories.^20^ These contradictory results reflected that the eighth T categories was not perfect enough and required further refinement. Previous study suggested that either pleural thickness or tumor volume measured by imaging equipment was predictive of survival.^38^ Additional studies focusing on the aforementioned parameters would probably lead to substantial refinement of T descriptors. The stage groupings have also been revised. We found that there was no significant survival difference in stage I versus II as well as stage II versus III. Owing to limited number of enrolled MPM patients, we did not subdivide stage I and stage III patients into stage IA/IB and stage IIIA/IIIB. In IASLC staging dataset, there was no significant separation between adjacent TNM stages, with the exception of stage II versus stage IIIA. To sum up, the current stage classification schema is less than satisfactory, and further prospective data collection and external validation would hopefully improve the staging of this rare malignancy.

With regard to gender, although previous population‐based studies from England and US identified gender as independent prognostic factors,^39^, ^40^, ^41^ this finding was not validated in this cohort of nonepithelioid MPM (Figure S2A–C) and previous studies of biphasic MPM.^21^, ^23^ Considering the relatively small numbers of nonepithelioid cases, we were not completely certain about the prognostic value of gender in nonepithelioid cases. To comprehend the impact of histologic subtypes on prognostic value of gender, it is necessary to look into variations in asbestos exposure, pathologic features, and sex hormones in future large population‐based studies. Since gender and N stage were prognostic factors only in epithelioid MPM rather than nonepithelioid MPM, one might wonder whether gender and N stage which were included in the nomogram could improve the survival prediction in biphasic MPM. After classifying biphasic patients into three groups: group A (N+/male), group B (N+/female or N0/male), and group C (N0/female), we found that group C demonstrated significantly better survival than group A (*p* = 0.026; Figure S3B), which indicated that combination of these two parameters could improve the survival prediction in biphasic MPM. A similar trend was also observed in epithelioid MPM (Figure S3A).

Several limitations of this study should be noted. First, absence of data on LNs station, operative technique, and imaging test limited further evaluation of disease involvement pattern. Second, the lack of information on resection margin, performance status, and complication could result in potential bias. Third, the administration of PORT and chemotherapy was not within a randomized study, and the treatment intention was not recorded in detail. Furthermore, this model evaluated only the survival benefit but not potential toxic effects for PORT candidates. We acknowledged that developing different nomograms for different histologic subtypes was a perfect solution, but limited number of nonepithelioid cases made this nearly impossible. Although the current nomogram was not the best model, it carried certain clinical value and provided reference for further research. Constant efforts on wider geographic data collection and incorporation of molecular tests are encouraged to improve this model.

In conclusion, the prognostic performance of eighth MPM TNM staging system was far from perfect after external validation using SEER database. Based on clinicopathologic factors, we developed a prediction model, which demonstrated significantly better discriminatory performance than eighth MPM TNM staging system. This novel prediction model could also help clinicians provide individualized estimation of survival benefit from PORT and establish optimal management and follow‐up strategy.

## AUTHOR CONTRIBUTIONS

**Yang Wo:** Conceptualization (equal); data curation (equal); formal analysis (equal); investigation (equal); methodology (equal). **Yizhou Peng:** Conceptualization (equal); data curation (equal); formal analysis (equal). **Zhigang Wu:** Methodology (equal); resources (equal). **Pengcheng Liu:** Software (equal); validation (equal). **Yan Shang:** Validation (equal). **Xuxia Shen:** Conceptualization (equal); validation (equal); visualization (equal); writing – review and editing (equal). **Yihua Sun:** Conceptualization (equal); formal analysis (equal); funding acquisition (equal); investigation (equal); supervision (equal); validation (equal); writing – review and editing (equal).

## CONFLICT OF INTEREST STATEMENT

None.

## ETHICS STATEMENT

Since the SEER database was publicly accessible, approval from Institutional Review Board was not required.

## Supporting information


Figure S1.

Figure S2.

Figure S3.

## Data Availability

Data are available upon reasonable request to the corresponding author.
