# Supplementary figures and images for: Individualized prediction of survival benefit from postoperative radiotherapy for patients with malignant pleural mesothelioma
Source: Cancer Med. 2023 Apr 19;12(11):12452–61. doi: 10.1002/cam4.5955 (PMC10278470; doi:10.1002/cam4.5955)

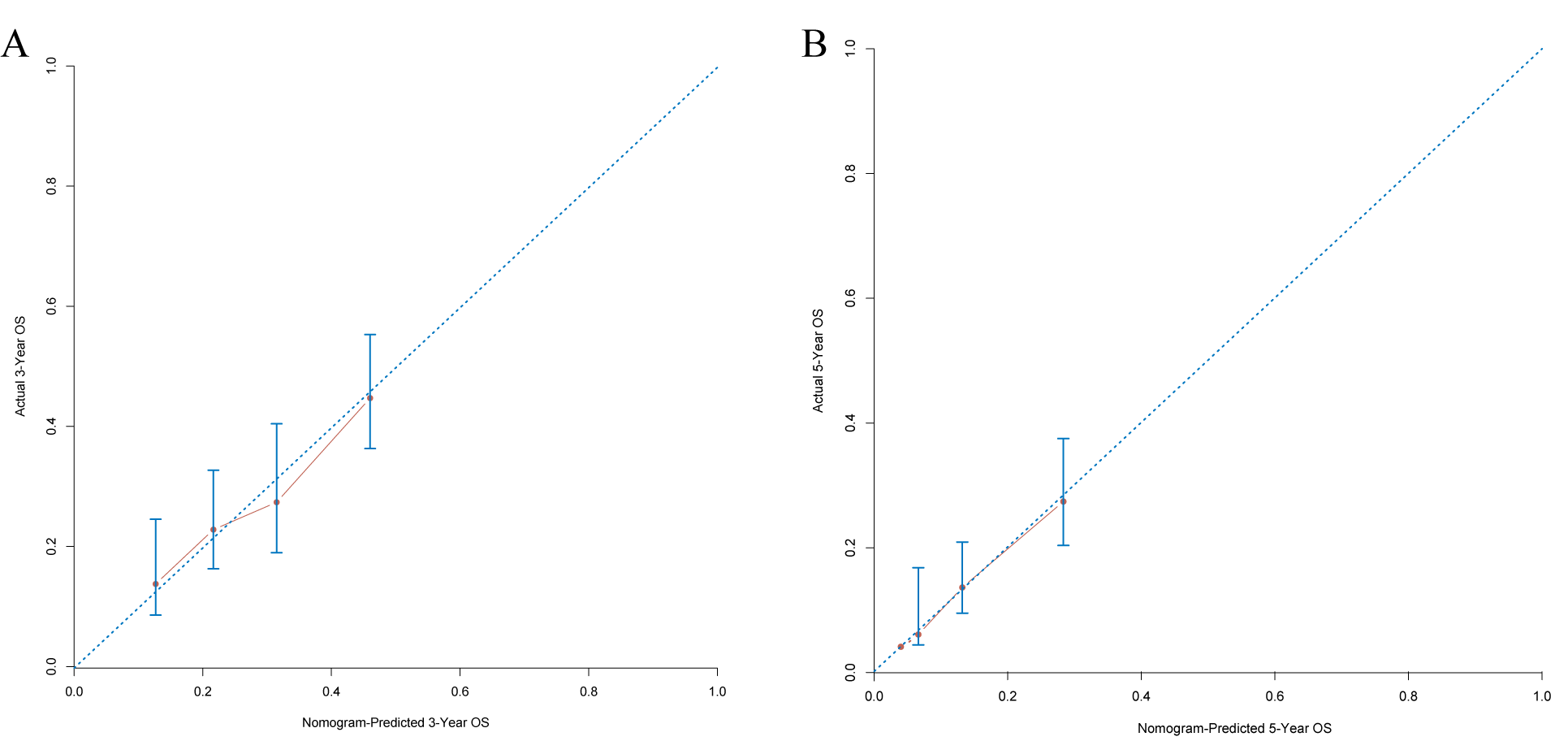

Supplement: Supplementary file 1 — Figure S1. [file CAM4-12-12452-s001.tif]

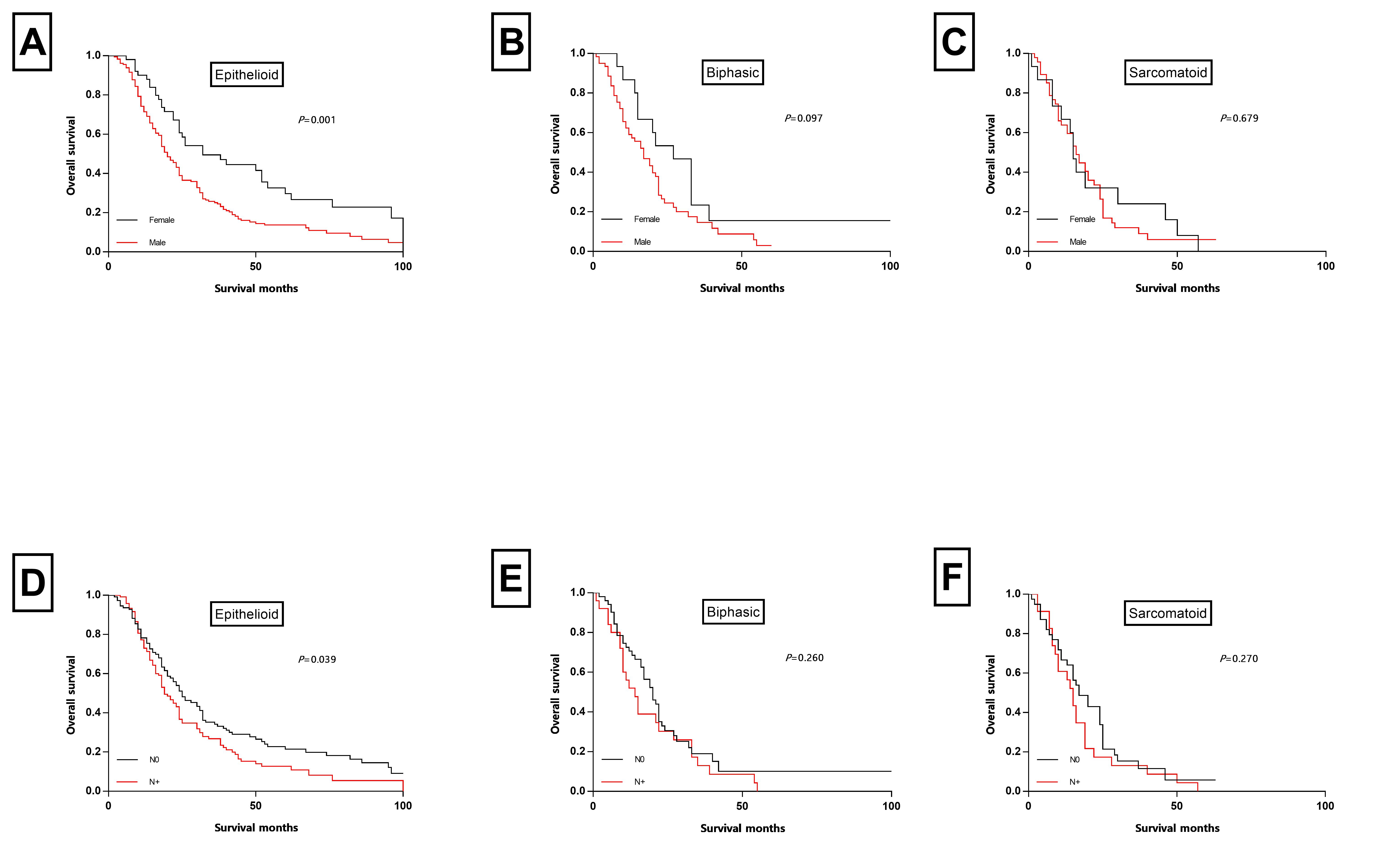

Supplement: Supplementary file 2 — Figure S2. [file CAM4-12-12452-s003.jpg]

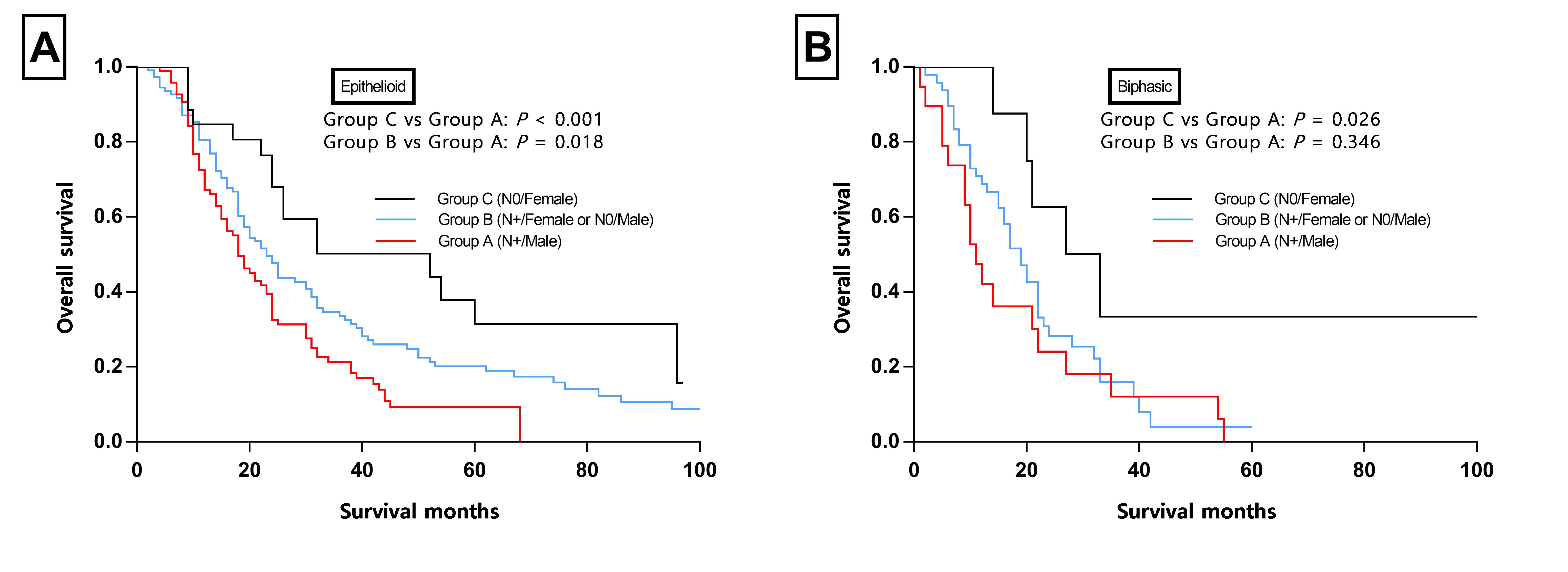

Supplement: Supplementary file 3 — Figure S3. [file CAM4-12-12452-s002.jpg]
